# Perfused Three-dimensional Organotypic Culture of Human Cancer Cells for Therapeutic Evaluation

**DOI:** 10.1038/s41598-017-09686-0

**Published:** 2017-08-25

**Authors:** Xiao Wan, Steven Ball, Frances Willenbrock, Shaoyang Yeh, Nikola Vlahov, Delia Koennig, Marcus Green, Graham Brown, Sanjeeva Jeyaretna, Zhaohui Li, Zhanfeng Cui, Hua Ye, Eric O’Neill

**Affiliations:** 1 CRUK/MRC Oxford Institute of Radiation Biology, University of Oxford, ORCRB Research Building, Roosevelt Drive, Headington, OX3 7DQ UK; 2 Oxford Instruments Nanoscience, Tubney Woods, Abingdon, Oxford OX13 5QX UK; 3 Institute of Biomedical Engineering, Department of Engineering Science, University of Oxford, Old Road Campus Research Building, Headington, Oxford OX3 7DQ UK; 4 Department of Neurosurgery, John Radcliffe Hospital, Headington, Oxford OX3 9DU UK

## Abstract

Pharmaceutical research requires pre-clinical testing of new therapeutics using both *in-vitro* and *in-vivo* models. However, the species specificity of non-human *in-vivo* models and the inadequate recapitulation of physiological conditions *in-vitro* are intrinsic weaknesses. Here we show that perfusion is a vital factor for engineered human tissues to recapitulate key aspects of the tumour microenvironment. Organotypic culture and human tumour explants were allowed to grow long-term (14–35 days) and phenotypic features of perfused microtumours compared with those in the static culture. Differentiation status and therapeutic responses were significantly different under perfusion, indicating a distinct biological response of cultures grown under static conditions. Furthermore, heterogeneous co-culture of tumour and endothelial cells demonstrated selective cell-killing under therapeutic perfusion versus episodic delivery. We present a perfused 3D microtumour culture platform that sustains a more physiological tissue state and increased viability for long-term analyses. This system has the potential to tackle the disadvantages inherit of conventional pharmaceutical models and is suitable for precision medicine screening of tumour explants, particularly in hard-to-treat cancer types such as brain cancer which suffer from a lack of clinical samples.

## Introduction

Although average patient survival after diagnosis of cancer is increasing, some hard-to-treat cancer types such as brain tumours still suffer from the lack of effective therapeutics. Meanwhile the pharmaceutical industry is in crisis with low success rates for compounds entering the clinical phase^1^. This situation is largely due to the limitations of the widely used *in-vitro* models and their inaccurate reflection of the clinical condition. Current *in-vitro* two-dimensional (2D) cell-based assays fail to recapitulate the complexity of the human tumour microenvironment, which includes three-dimensional (3D) anatomic structures of the extracellular matrix (ECM) and cellular heterogeneity reflective of the tumour niche^2^. In addition, tumour and supporting tissues have high plasticity under mechanical stimuli imposed by the fluidic phase of the microenvironment, a phase which includes the microcirculation of capillaries and lymph, and interstitial flow^3^. Interstitial flow provides mechanical clues for tissue remodelling, by manipulating the cell-ECM and intercellular interactions in a 3D *in-vivo* environment^4^. Recently, models which recapitulate the tumour microenvironment, such as 3D co-culture have been established and are beginning to be used in the re-emergence of phenotypic screening^5, 6^. These models include the organotypic culture of cancer cells, integrating heterogeneous cell types and 3D scaffolds with microarchitectures in order to increase the physiological relevance of the models^7^. In addition, scaffold systems such as hydrogel can provide a wide range of physical and chemical properties and can therefore be optimised for various cell types^8^. Nevertheless, an important part of the tumour microenvironment, the flow originating from the microcirculation perfusion has been relatively ignored^8, 9^. The contribution of flow can be separated into two components: shear stress and nutrient supply, however, most perfusion strategies designed to replicate the *in-vivo* microcirculation so far have been mainly focused on how fluid dynamics influences tumour growth or cancer cell migration^2, 7, 8^.

Recently, evidence from basic and clinical oncology reveals the importance of the selection and development of cancer cell subpopulations with different pluripotency in tumour progression. A rare subpopulation of these cells has a development programme resembling that of stem cells, including self-renewable ability, promoted proliferation, and capacity for quiescence, enabling resistance to traditional chemotherapy targeted at rapidly dividing cells^10–12^. Stem cell markers such as SOX2 and Nanog were found in neuroblastoma tissue^10, 11^, and the expression of neuronal progenitor markers like GFAP is frequently observed in glioblastoma^10, 11^. It is widely accepted that mechanical cues from the shear stress arising from blood circulation or interstitial flow are vital in tissue development and stem cell biology^13, 14^, but so far there is limited information on how mechanical cues remodel the tumour microenvironment and influence self-renewal, tumour maintenance and resistance to anti-cancer therapeutics. Similarly, the role of stress as a result of nutrient depletion in the development of stem cell like traits in tumours is as yet unclear.

In this study, we examine the effect of continually replenishing nutrient supply on the differentiation state of 3D cultures of various tumour cells. Three representative engineered tissue models (spheroids, hydrogel sandwich and embedded) were cultured in perfusion bioreactors to model the effect of physiological conditions on cancer cell growth and response to anti-cancer therapeutics for up to 35 days. We demonstrate that static cultures display increased indicators of cell stress and altered therapeutic responses compared to the more physiologically relevant perfused cultures.

## Results

### Characterisation of the flow dynamics in the perfused bioreactor

**Figure 1:**
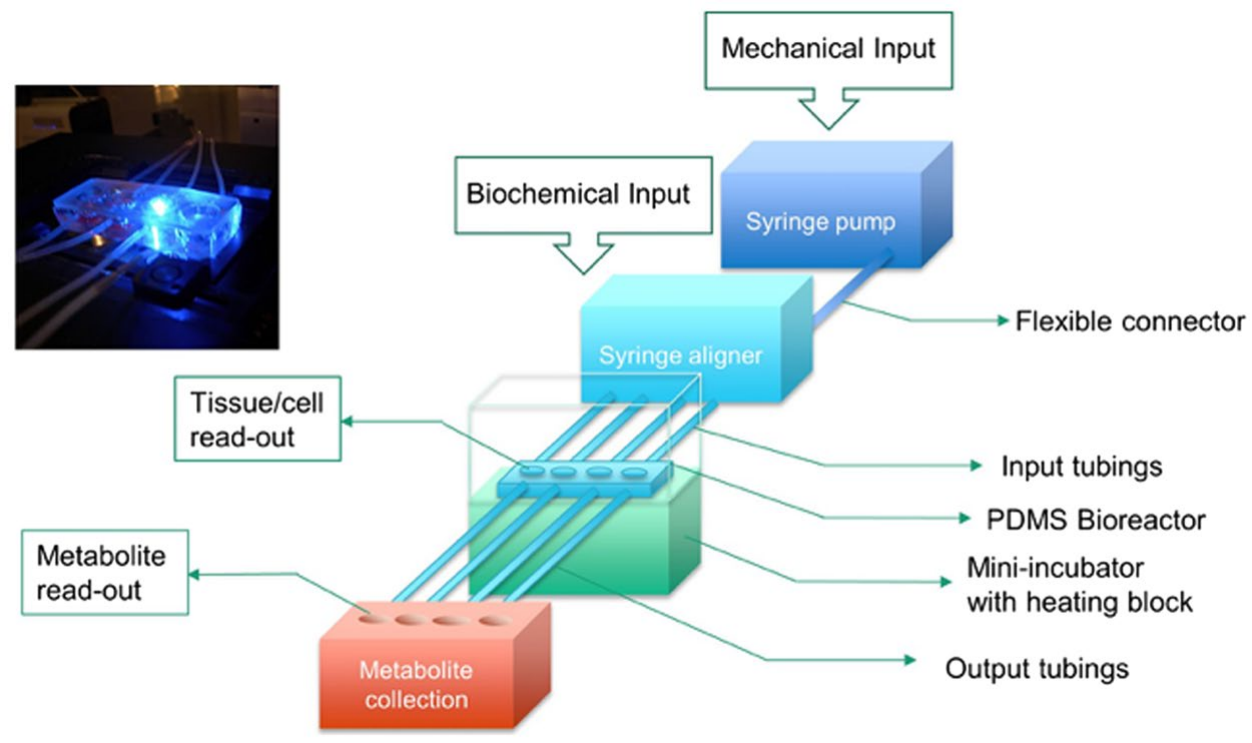
Desktop bioreactor platform for perfused microtumour culture. Syringe pumps are used to provide a continuous flow which allows a consistent nutrient supply to the Bioreactor. A syringe aligner allows delivery of therapeutics to the flow. The bioreactor can be mounted on a normal fluorescence microscope (upper left corner photograph) for real-time imaging. The design of the upstream input and downstream analysis is highly modular which allows other users to integrate their own apparatus into the design.

**Figure 2:**
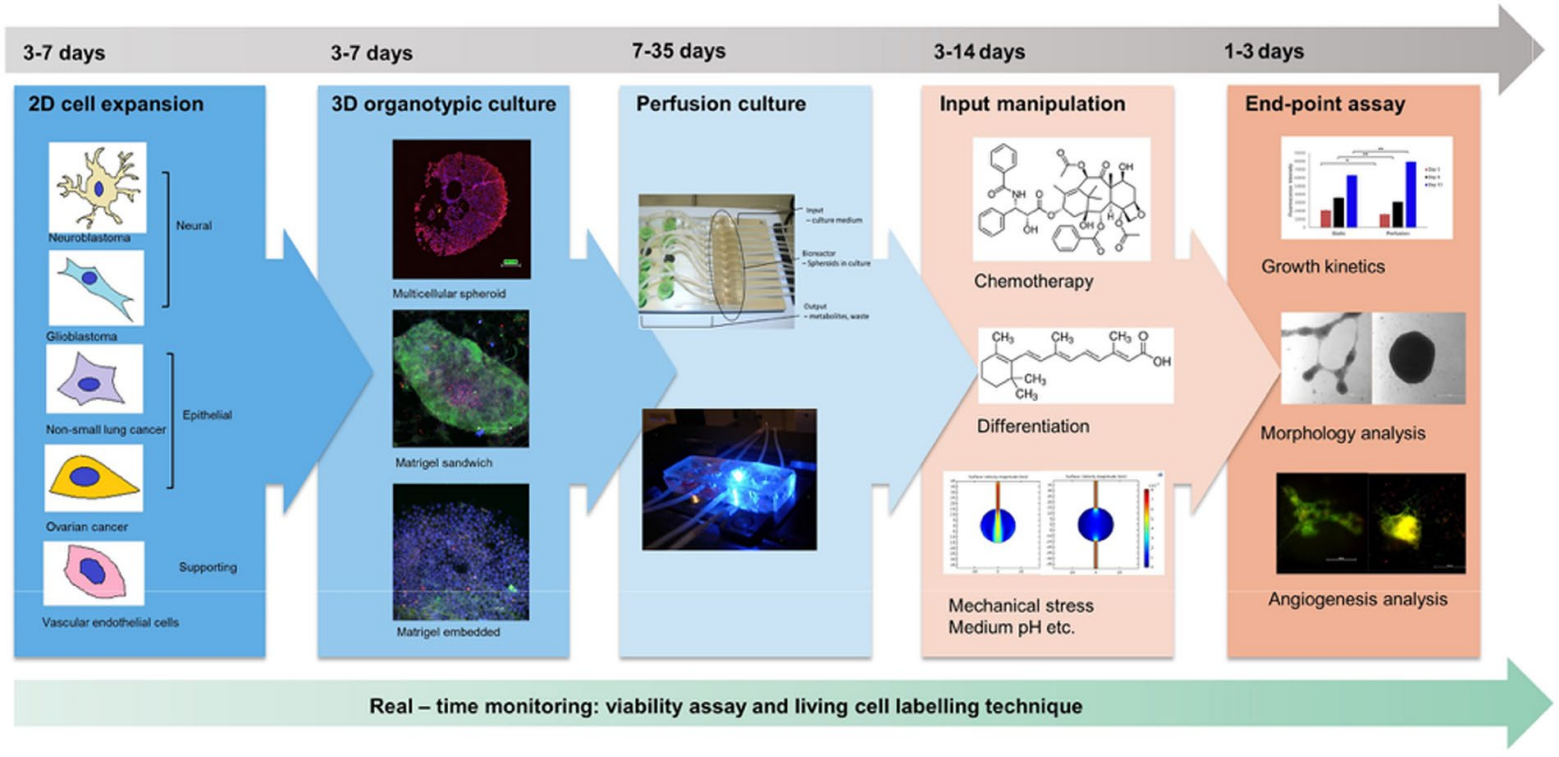
Schematic workflow showing the step-by-step process to stabilise microtumours for long periods by utilising our perfusion system. Also indicated is the process for introduction of anti-cancer therapeutics for evaluation.

**Figure 3:**
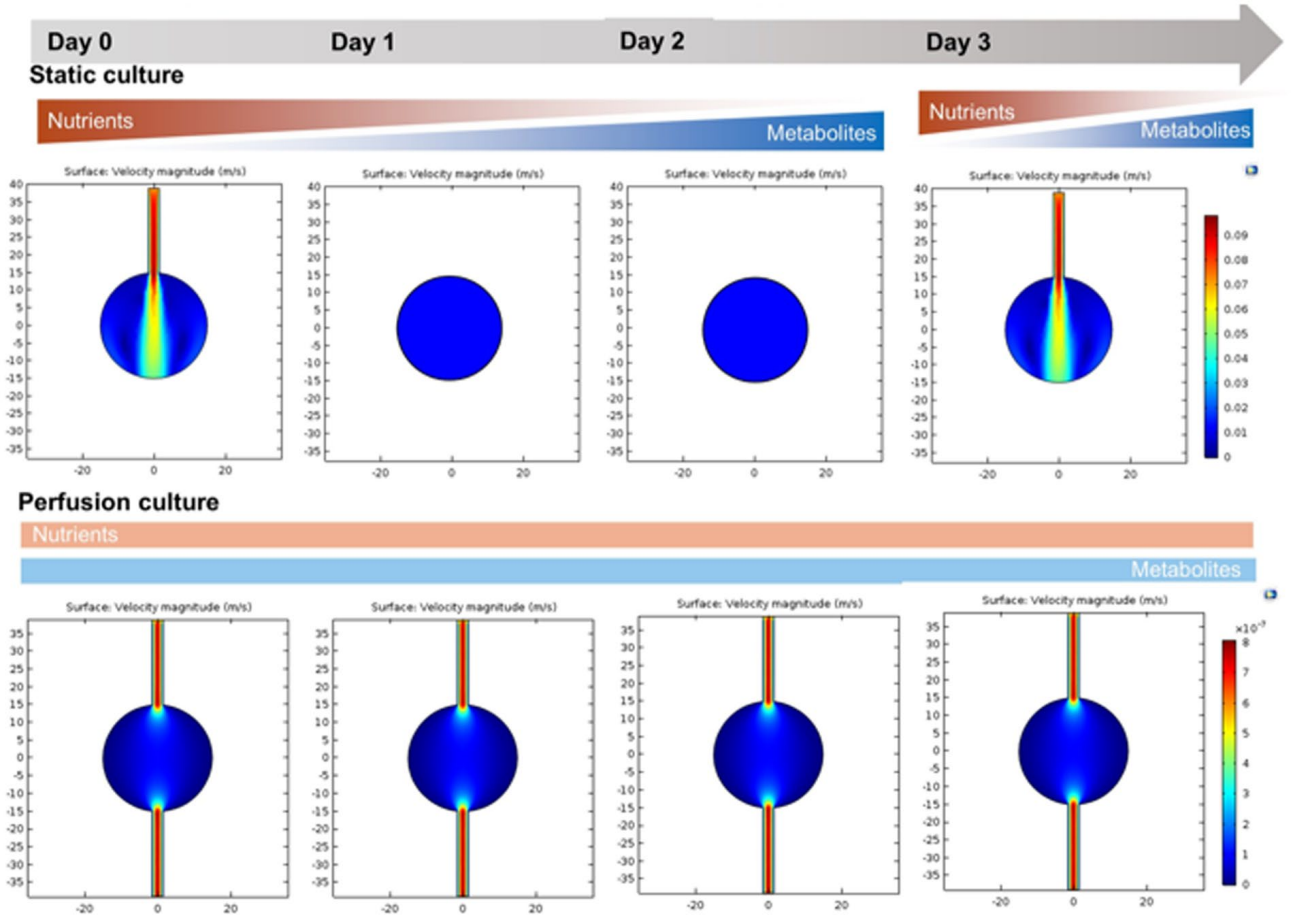
Velocity simulation inside one well of the bioreactors. Static culture experiences the fluctuation of mechanical environment through the frequent medium change. In the heat map shift to the red side of the colour scale indicates a higher flow velocity. Perfusion culture improves the homeostasis of the culture environment, especially over long-term culture.

We previously reported a perfused bioreactor that permitted assessment of the growth of cancer cells in 3D for up to 17 days^15^. We have further adapted this model to allow a ‘desktop’ design for longer term longitudinal studies (<35 days) that includes of a syringe aligner for delivery of additional biochemical inputs (Fig. 1). Bioreactor components have been designed to be mounted on a normal fluorescence microscope for real-time imaging of long-term cultures to allow greater phenotypic evaluation of tumour responses to therapeutics. Our workflow further develops current 2D and 3D cell culture platforms to allow growth of cancer cells to larger organotypic culture or ‘microtumours’ that more closely replicate microenvironmental conditions in human tumours (Fig. 2). Moreover, this set-up allows the concurrent delivery of therapeutics and monitoring of cell populations in real-time using fluorescent labelling of cells. One of the main difficulties with long-term cell culture is availability of nutrients and build-up of cellular metabolites over time. Episodic replenishment of media to static cultures supports growth by replacing nutrients, however this presents large variations in supply and also generates mechanical stress that perturbs maintenance over long periods and can be prevented by continual perfusion of nutrients. In order to model the effects of mechanic force and nutrient supply on perfused versus static cultures, a velocity field simulation was performed using COMSOL multi-physics platform. Briefly, it was assumed that in static culture, 500 µL medium was added into each well of a standard 24-well plate in two minutes, and the culture was maintained for 3 days (72 hours) until day 4, when spent medium was replaced with fresh; while in perfusion culture the identical volume of culture medium (500 µL) was perfused over 72 hours at a constant rate 6.94 µL/hr. Assuming all the nutrients are highly soluble in the medium, the velocity field distribution reflects the distribution of the nutrients supply throughout the time course of the culture. As shown in Fig. 3, COMSOL multi-physics simulation reveals that the perfused culture has a consistent velocity field distribution over a culture well, while in the standard ‘static culture’ the cultured tissue will experience cyclic non-physiological stresses such as high levels of growth factors, metabolites and thermal shock.

### Perfused Matrigel ‘sandwich’ cultures prolong the maintenance of neuroblastoma microtumours

**Figure 4:**
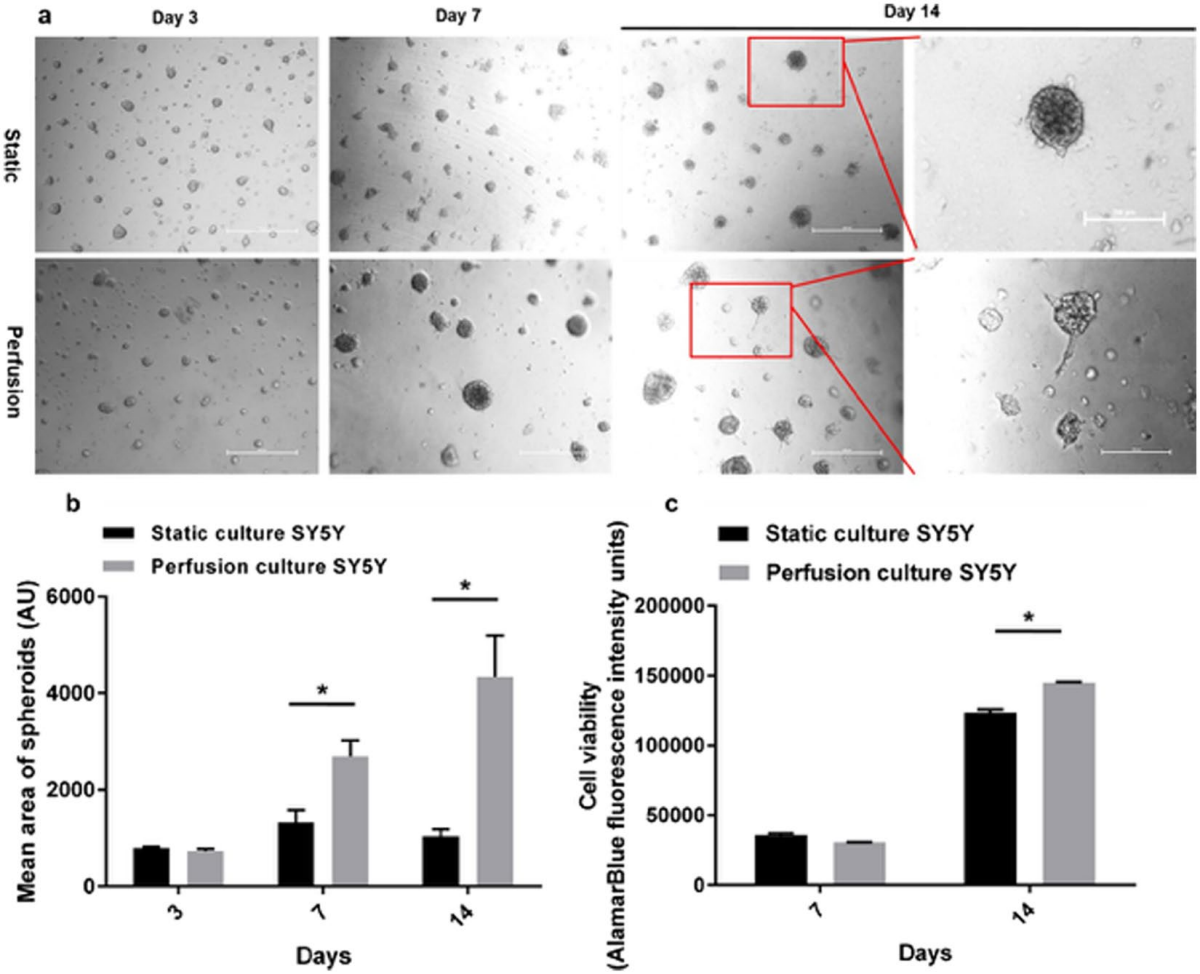
Perfusion significantly influenced the morphology of neuroblastoma spheroids originated from SY5Y in long-term culture. (**a**) Representative real-time imaging of neuroblastoma cells SY5Y cultured in Matrigel sandwich comparing the static and perfusion culture over 14 days of culture. Scale bar: 500 µm. (**b**) Image analysis using ImageJ reveals the significant growth difference at day 14, between static and perfusion culture. Mean spheroid areas were compared as representative parameters. (**c**) Tissue metabolic viability shows that at day 14 perfusion culture has significantly higher viability compared with static culture. For statistics, all the groups have four replicates and the experiments were repeated for three times. Results are presented as mean ± SD. *p < 0.05 by Student’s t-test.

**Figure 5:**
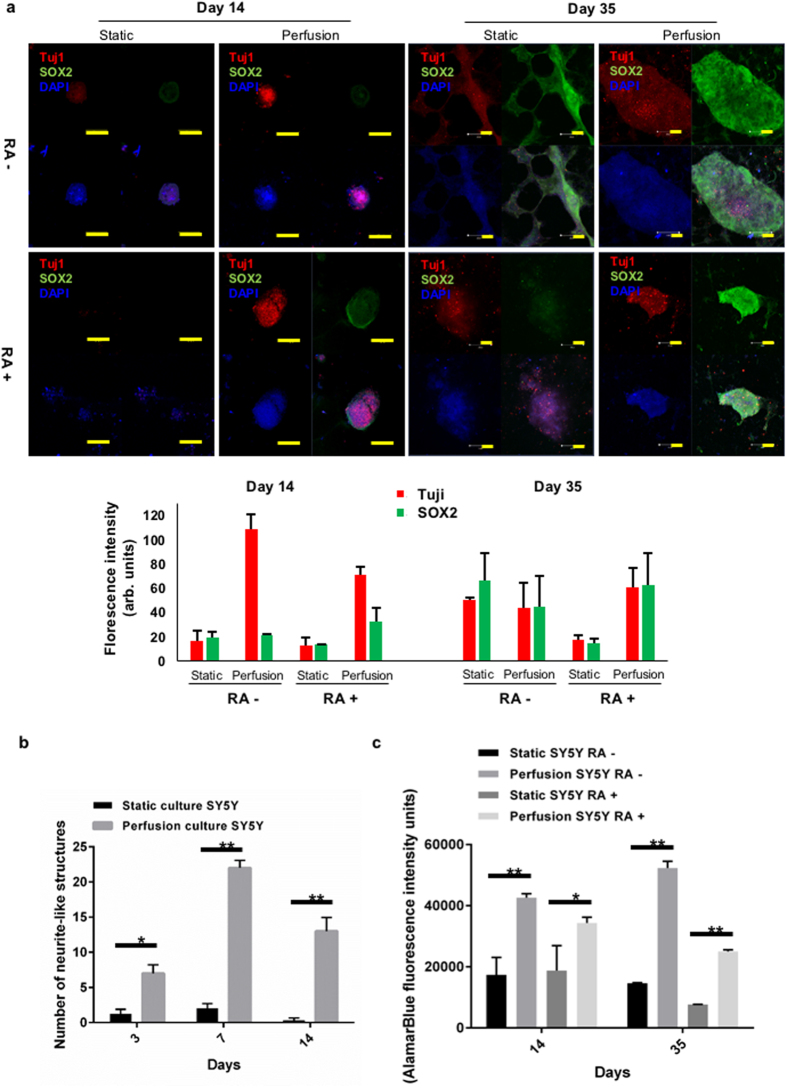
Neuroblastoma microtumours in Matrigel sandwich mimicking differentiation therapy. (**a**) Perfusion altered the pluripotency markers of the micro-neuroblastoma, shown at day 14. Scale bar: 100 µm. Comparisons between micro-neuroblastoma in the static and perfusion culture, at day 35. Quantification of fluorescent images by ImageJ shown in bar graph. (**b**) Image analysis via ImageJ reveals that in perfusion culture more neurite-like structures were observed, indicating a potential differentiation – promotion effect of the perfusion flow. (**c**) Retinoic acid treated group in perfusion has lower treatment response compared with the static culture, indicating a higher resistance by perfused neuroblastoma microtumours to the differentiation therapy mimicked. For statistics, all the groups have four replicates and the experiments were repeated for three times. Results are presented as mean ± SD. *p < 0.05; **p < 0.01 by Student’s t-test.

We previously demonstrated perfusion can increase cell viability of free floating colorectal spheroids^15^. To assess further the reliability of this system as a true model we chose to include the physical forces that would be imparted by the ECM and also determine the effect on cellular phenotypic heterogeneity, characteristic of most cancers. As observed for colorectal^15^ and lung cancer spheroids (Fig. s1), neuroblastoma SY5Y 3D cultures embedded in matrigel ‘sandwich’ (to mimic ECM) indicate that static cultures display limited growth and consistently feature a necrotic core in the central area of the 3D tissue after long-term culture (>7 days). However, 3D SY5Y microtumours in perfused culture showed increased tissue viability (using metabolic activity as a proxy measurement for viability) and volume compared with the culture in conventional static environment (Fig. 4a,b and c). Interestingly, at day 14 neurite-like extensions could be observed emanating from perfused microtumours, while in the static culture no such structures were observed. As this indicated a shift in cellular phenotype, microtumours were fixed and subjected to immunofluorescence staining for pluripotency marker SRY (sex-determining region Y) – box 2 (SOX2) (green), and neuronal differentiation marker class III beta-tubulin (Tuj1) (red) to characterise the extent of phenotypic plasticity and/or differentiation. Notably, colonies could grow to an equivalent size under both conditions up to day 14 (100  um). When cultures grew beyond 500 um (day 35), perfused cultures maintained round tight colonies whereas these where not supported under static conditions and more diffuse cell packing appeared to be predominant (Fig. 5a). The presence of pluripotent cells (SOX2^+ve^) were observed under both conditions but only evident at day 35. Interestingly, only perfused cultures displayed expression of Tuj1 at day 14, although both cultures did so at day 35 indicating a potential shift towards differentiation into mature neurons earlier under perfusion (Fig. 5a,b). Neuroblastomas are treated with retinoic acid (RA), which is a clinically approved therapy aimed at inducing stem cell like tumour cells to differentiate and thus be more susceptible to treatment. One main advantage of perfused cultures presented here is that we can deliver therapeutics in a more physiologically appropriate manner and replicative of how tumours would be exposed to therapeutics *in vivo*. As shown in Fig. 5a, RA resulted in the disappearance of the SOX2^+ve^ population from static cultures, in line with stimulation of differentiation. Strikingly, under perfusion we observed the maintenance of the more stem-cell like population at day 35 and the appearance of SOX2^+ve^ cells at day 14, which were not observed in the absence of RA (Fig. 5a). Moreover, under perfusion RA treatment appeared to limit viability along with maintaining a more compact colony shape (Fig. 5a,c), which could be due to greater physiological relevance as secretion of ECM is known to support contraction of cells and may explain the difference in colony shape and size. The presence of both Tuji^+ve^ and SOX2^+ve^ cells is in line with clinical observations of tumour heterogeneity including stem-cell like populations in neuroblastoma that are thought to be responsible for therapeutic resistance and demonstrates the greater physiological relevance of perfused cultures. In line with this, normalising to the viability of the corresponding control group, perfused micro-neuroblastomas were more resistant to therapy (Fig. 5c). Interestingly, SY5Y cells.

### Greater neurite-like protrusion are observed in HUVEC-neuroblastoma microtumours under flow

**Figure 6:**
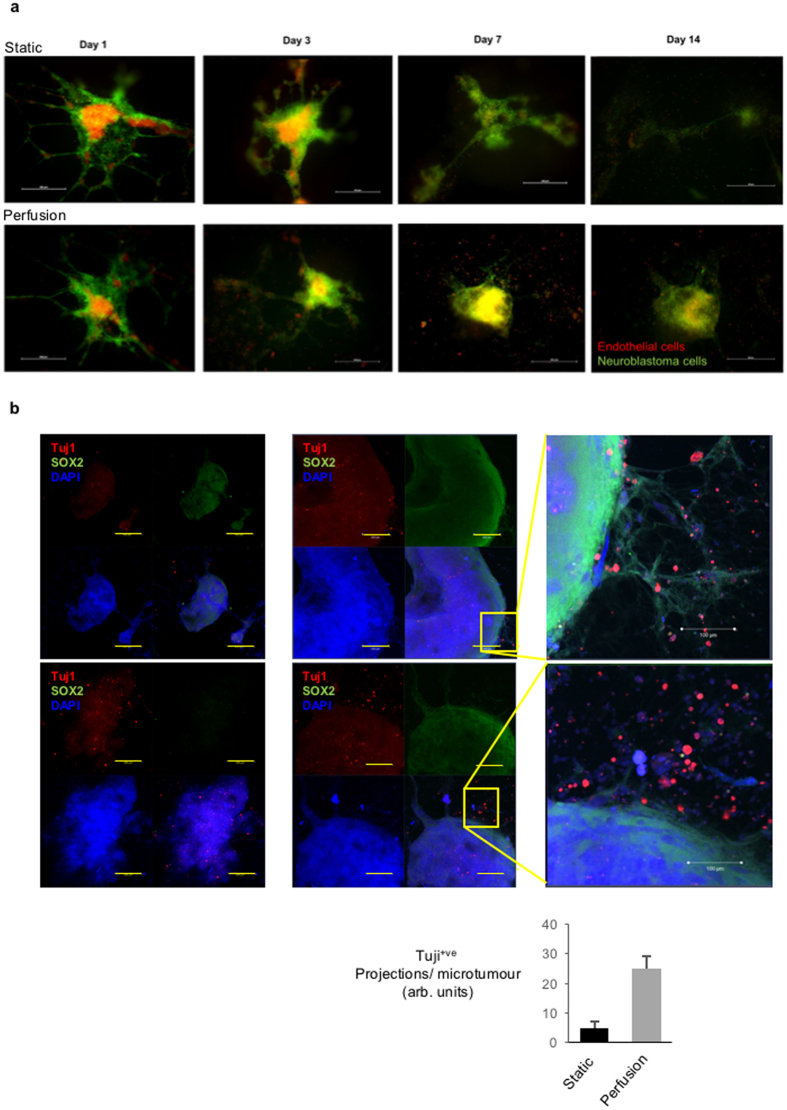
Co-culture of vascular endothelial cells HUVECs with neuroblastoma microtumours from SY5Y in the static and perfusion culture. (**a**) Perfusion stabilised the micro-structure of neuroblastoma – endothelium co-culture over 14 days. Scale bar: 500 µm. (**b**) Perfusion increased the Tuj1 positively stained neurite structures in neuroblastoma SY5Y co-cultured with vascular endothelial cells HUVECs at day 35. Scale bar: 200 µm. Quantification of Tuji^+ve^ fluorescent cell pretrusions by ImageJ shown in bar graph.

Angiogenesis is widely accepted as a key step in tumour development and vital part of the tumour microenvironment^16, 17^. Human umbilical vein endothelial cells (HUVECs) form endothelial like structures that can mimic angiogenesis and we find that these readily form independent of static or perfused conditions, but display slightly more stable branched structures under perfusion <14days (Fig. s2). To approach a replica of the *in-vivo* tumour microenvironment and supporting stroma, we next co-cultured vascular endothelial cells with the neuroblastoma microtumours. As shown in Fig. 6a and Fig. s3a, vascular endothelial cells (HUVECs, red) were co-cultured with neuroblastoma cells (SY5Y, green) in Matrigel. An organised co-localisation of endothelial and neuroblastoma microtumour tissue was observed under both conditions at day 1, but by day 7 HUVECs were only maintained in perfusion culture. By day 14, large co-cultures were maintained under perfusion with equivalent cell populations, whereas in the static culture SY5Y monocultures were of more limited size (Figs 6a, s3b). Surprisingly, the formation of endothelial like structures by HUVECs was lost upon co-culturing with tumour cells in longer term cultures. To investigate if perfusion has an impact on the development status of the co-cultured microtumours, we again stained pluripotency transcription factor SOX2 (green) and differentiation marker Tuj1 (red) (Fig. 6b). As observed under monoculture at day 35 (Fig. 5a), the static cultures preferentially lost the SOX2^+ve^ population and perfused cultures display more evidence of Tuji^+ve^ neuron-like cells in the presence of RA (Fig. 6b). Interestingly in the presence of RA, SY5Y neuroblastoma cells appeared to in the presence of endothelial cells (Figs 5a and 6b).

### Ovarian cancer cells are more susceptible to paclitaxel in co-cultures under perfusion

**Figure 7:**
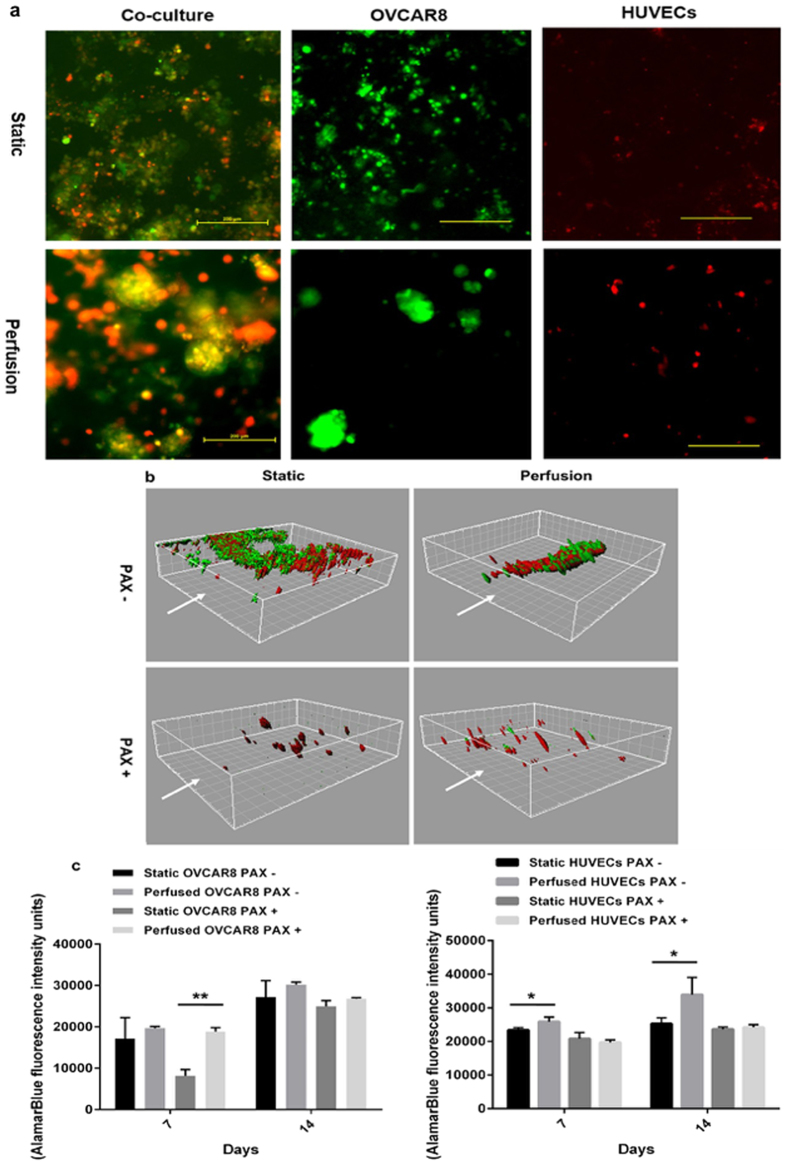
Co-culture ovarian microtumours in the static and perfusion culture. (**a**) Ovarian cancer cells OVCAR8 co-cultured with vascular endothelial cells HUVECs in Matrigel sandwich for 14 days in the static or perfusion culture, with OVCAR8 only or HUVECs only as controls. Scale bar: 200 µm. (**b**) Testing Paclitaxel (PAX) chemotherapy using ovarian cancer cells OVCAR8 co-cultured with vascular endothelial cells HUVECs in the static or perfusion culture; 3D reconstruction of MPM images via Imaris at day 14 of treatment. White arrows indicate the flow direction. Grid unit: 20 µm. (**c**) Tissue viability inhibition measured by AlamarBlue assay, showing the influence of perfusion on OVCAR8 microtumours, HUVECs, and co-culture of OVCAR8 and HUVECs microtumours in sandwich Matrigel. For statistics, all the groups have four replicates and the experiments were repeated for three times. Results are presented as mean ± SD. *p < 0.05; **p < 0.01 by Student’s t-test.

We further tested the feasibility of co-culture of endothelial cells with a cancer cell line of epithelial origin, the ovarian cancer derived OVCAR8. As shown in Fig. 7a, at day 14 OVCAR8 microtumours in Matrigel showed an improved integrity in perfusion culture, indicated by larger 3D spheroid structures, whereas static culture microtumours were mostly degraded, while cells maintained viability and were not apoptotic (Fig. s4). Classical tubule branching structure of endothelial cells (HUVECs) in 2D was not observed when cultured in Matrigel sandwich either in static or perfusion culture. As observed with SY5Y:HUVECs above, OVCAR8 (green) and HUVECs (red) co-cultures displayed significantly improved maintenance under perfusion at day 14 (Fig. 7a). Z-stack reconstruction of co-culture ovarian microtumours also indicated that statically cultured ovarian microtumours are disordered whereas perfused co-culture microtumours have a discernible order 3D architecture with the heterogeneous cell populations in close association (Fig. 7b). We next evaluated whether these effects on cell-cell interaction within the co-culture impacted on therapeutic responses to chemotherapy. Paclitaxel (PAX) is a common treatment for ovarian cancer and as shown in Fig. 7b, 14 days of treatment with perfused PAX versus episodic treatment in static microtumours was sufficient to ablate the majority of ovarian cancer spheroids (green). However, in static cultures a significant number of ovarian cancer cells persisted that appear to be closely associated with the endothelial cells (red), while in perfused cultures PAX eradicated the cancer cells but left normal HUVECs intact. This suggests a potential efficient screening platform where tumour cell sensitivity and normal tissue toxicity could be assessed simultaneously and under physiologically relevant conditions.

### Perfusion improves the maintenance of glioblastoma microtumours embedded in Matrigel

**Figure 8:**
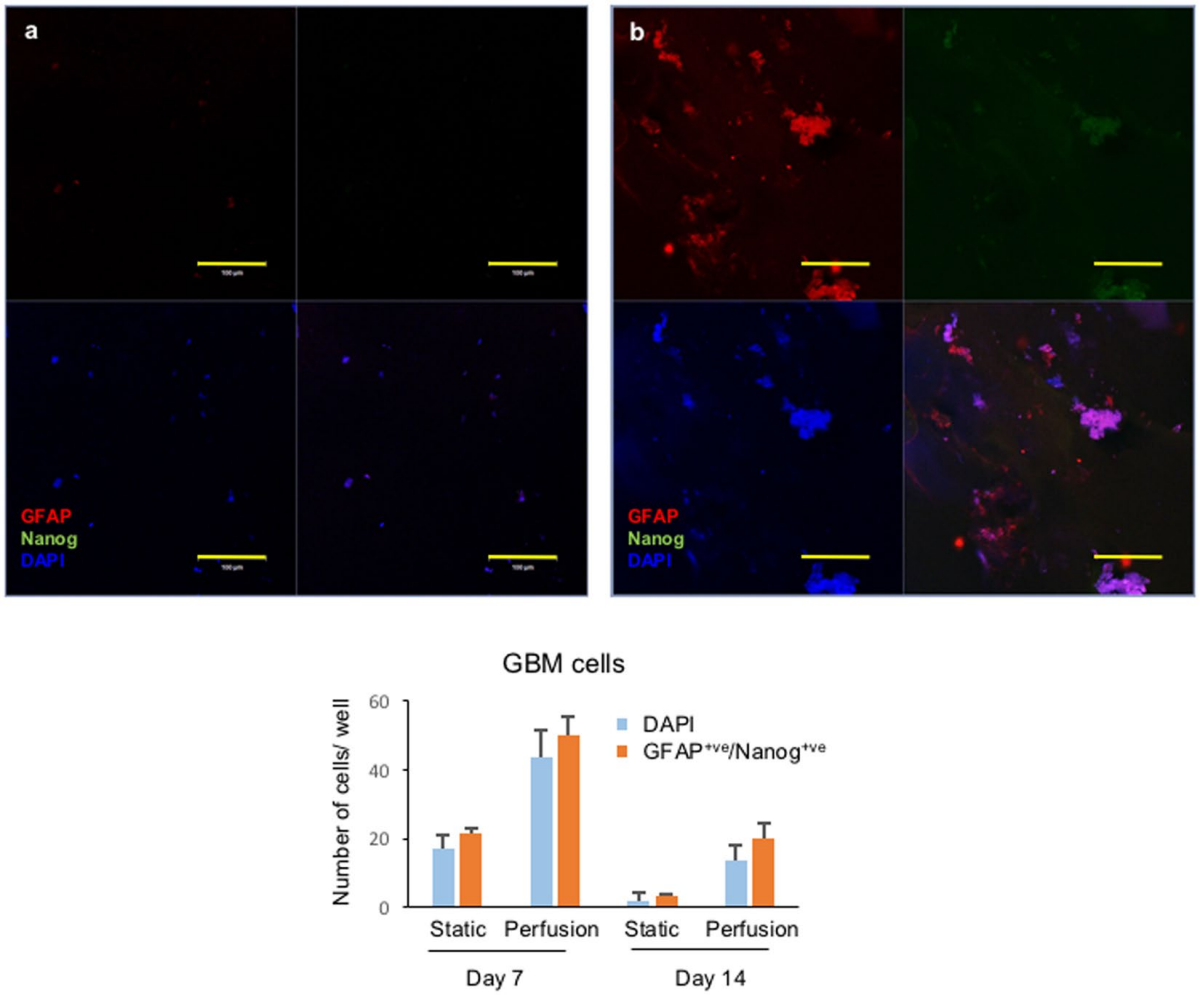
GBM microtumours embedded in Matrigel, formed by primary cells isolated from a clinical sample. (**a**) Static culture of GBM microtumours. (**b**) Perfusion culture of GBM microtumours. GBM microtumours were formed by embedding primary cells in Matrigel, and then the 3D structures were extracted as described in Materials and Methods, followed by staining with astrocyte differentiation marker glial fibrillary acidic protein (GFAP, red fluorescence) and pluripotency marker Nanog (green fluorescence). Scale bar: 100 µm. Quantification of fluorescent cells shown in bar graph.

GBM is the commonest malignant brain tumour in adults, and the aggressiveness of the symptoms together with a lack of physiologically relevant pre-clinical models makes it urgent to develop effective therapeutic evaluation models for the disease. Maintenance of tumour tissue or derivation of cell lines from GBM is notoriously difficult^18^, therefore we wanted to determine whether perfusion may assist in developing organotypic cultures. As a first step we examined glioblastoma cell line GAMG under both cell culture systems for levels of Nanog (green), a marker of pluripotency and found at high levels in the nucleus of GBM cancer stem cells^11^, and glial fibrillary acidic protein (GFAP) which is an astrocyte marker of differentiation. Static cultures produced unusual staining for both markers, however only perfused culture demonstrated high levels of nuclear Nanog (Fig. s5), as observed for SOX2^+ve^ population in Neuroblastoma and Ovarian cancer cells (Figs 5 and 6). Both static and perfusion cultures could be seen to support heterogenous populations as evidenced by GFAP and Nanog staining (Fig. s5). GAMG cells, like all other cell lines, have been selected for their ability to grown in 2D tissue culture conditions and questions remain over their reliability in reflecting real tumours. We next attempted to apply the perfused bioreactor to primary glioblastoma cells isolated from clinical samples. Primary GBM cells did not appear to grow under static culture conditions, in keeping with known difficulties, as indicated by the low fluorescence signals shown in Fig. 8. However, under perfusion, primary GBM cells were not only maintained but demonstrated stable expression of GFAP and a portion of Nanog^+ve^ that would be expected of primary tumours *in vivo*. In summary, the results shown in Fig. 8 demonstrated that 3D microtumours consisting of primary GBM tissue can be grown in Matrigel under perfused conditions.

## Discussion

Perfusion bioreactors have been developed primarily to promote homogeneity within cell cultures in order to optimise protein production. Recently, the opportunity to adapt these technologies to culture micro-tissue and cell spheroids *in-situ* has begun to be appreciated. In this study we applied a bioreactor design that we previously optimised for 3D culture in which the flow rate used ensured that the total nutrient supply is the same in both cases with minimal shear stress, as opposed to the cyclical process in static cultures in which nutrients are replaced every few days. We designed an extremely low perfusion flow (<10 µL/hr), to provide comparable nutrient supply to both the perfusion and static cultures and allow an objective comparison. The shear stress under this low perfusion rate will be limited, which can also be viewed in the velocity field simulation (Fig. 3). Nevertheless, we considered that there would be a mixture of laminar flow and turbulent flow in the bioreactor wells, in contrast to most main-stream bioreactors used in tissue engineering which have mostly turbulent flow to ensure the homogenous production of the desired products. However, most of these agitated bioreactor models focus on protein production rather than the physiological relevance of the cultured microtissues and therefore provide relatively randomised development patterns in the cultured tissues^19, 20^. In addition, the laminar flow is dominant in transportation and dynamics of microcirculation. In our point of view as well as suggested by other researchers, it is necessary to consider the physiological relevance when transferring some of the concepts and principles from industry-scale bioreactors into bench level biomedical research^8, 21^. This is the main reason we designed this low perfusion rate, with laminar flow – directed mass transfer being the dominant in the perfusion culture. Here we utilised a panel of cell types, cultured in three most commonly used 3D culture formats (spheroids, hydrogel sandwich and embedded), to give a demonstration of how perfusion can improve the cell viability and morphological integrity over long-term culture. Morphologically, there were significant differences between static and perfused cultures. For each cell line and under each condition the perfused cultures had a greater viability and formed more defined structures.

In addition to direct observation on morphology, we also confirmed the greater degree of differentiation in perfused tumours by monitoring differentiation and pluripotency markers. Differentiation status can provide clues on the stress-level experienced by the culture tissues. Various stress factors, including nutrition and oxygen, can induce de-differentiation^22–25^. Furthermore, cancer cells with increased pluripotent potential or ‘stem-cell’ properties are more resistant to chemotherapy, which is an emerging hypothesis to explain therapeutic resistance and cancer relapse. For instance, increased levels of Nanog can be correlated to tumour stem cells in GBM and indicate the survival and malignancy of the disease^26^. Moreover, SOX2 can reprogram differentiated cells into pluripotent cells in concert with other factors and is overexpressed in various cancers^27^. Therefore, not surprisingly, it has been suggested that targeting pluripotency transcription factors such as Nanog and SOX2 can be a promising strategy to solve the current therapeutic limitation on hard-to-treat cancer types such as GBM^28^. Differentiation markers such as β-tubulin III was correlated with resistance to DNA-damaging and microtubule-interrupting chemotherapy^29, 30^; increased GFAP levels have been correlated with tumour volume, tumour necrosis among the GBM patients^31^. This is also the basis for differentiation induction therapy such as the retinoic acid treatment we mimicked in this study (Figs 4 and 5). Clinically, retinoic acid can be used twice per week, for two weeks after high-dose chemotherapy and stem cell transplantation^32^. Reports suggest that retinoic acid acts as pro-differentiation agent which promotes the cancer cells differentiate into a more normal cell like phenotype and causes growth arrest^33^. It is intriguing therefore that in our neuroblastoma system the cells in the perfused cultures are more resistant to treatment with retinoic acid or paclitaxel, despite having a higher proportion of differentiated cells. We also noticed that in Fig. 5a in the perfused microtumours the RA has the opposite effect, though the exact mechanism still remains unclear.

To mimic tumour tissue microenvironment in a perfused system is obtaining increasing attention recently. Most studies on tumour microenvironment are usually carried out in a petri-dish in the absence of flow, though in the *in-vivo* reality these intercellular and cell-ECM interactions actually happen in a fluidic environment, regulated by biological, chemical and physical signals conducted via vascular microcirculation and interstitial fluid^3, 4, 8, 9, 34, 35^. This dynamic fluidic phase includes interstitial flow, blood and lymph perfusion. In this study we focused mainly on the tumour angiogenesis by integrating angiogenesis-leading cell type vascular endothelial cells into our neuroblastoma cells SY5Y and ovarian cells OVCAR8 microtumour models. We noticed that although tubule structures were observed in the angiogenesis co-culture, the tubule network degraded after 7 days (Fig. 6). To solve this issue, future work will combine other supporting cell types in order to increase the physiological relevance of the system, such as fibroblast and pericyctes, with the hypothesis that an increased level of *in-vivo* relevance will increase *in-vitro* tissue morphology maintenance. We are also aware that Matrigel is a hydrogel with complex components, with relatively weak mechanical strength, and degrades significantly over long-term culture. Nevertheless, we used Matrigel for this study because that the biologically relevant components in the Matrigel, such as laminin, are vital for morphology and function maintenance of cultured tissues, and growth of many organoids^20^. For our future study hydrogel scaffolds are already being designed that combine artificial materials with Matrigel to allow a controlled microcirculation initially but later degradation of artificial materials should allow the integration of microtumour tissue with the supporting structures.

In this study, all 3D structures showed heterogeneity: even in the simplest 3D culture format, multicellular spheroids (Fig. s1). Precisely where stem cell like cancer cells reside in the tumour tissue and whether we can recreate them *in-vitro* to assess therapies remains an open question^10, 11, 28^. Regarding to the pluripotency/differentiation markers, we show that the distribution of cell populations in 3D hydrogel culture varies depending on whether the cultures are static or perfused, and that this influences the systems’ responses to certain therapeutics. We propose that applying a designed perfusion flow 3D bioreactor, together with the observation on a panel of pluripotency/differentiation markers would be an effective approach to replicate the differentiation status of 3D culture *in-vitro* tissues and is likely to highlight more physiologically relevant responses to therapeutic testing.

In summary, this work developed controlled perfused three-dimensional tumour models, including intercellular 3D structure (multicellular spheroid model), extracellular matrix (Matrigel sandwich and embedded models) and supporting cells (angiogenesis). The workflow of creating microtissues in the bioreactors was optimised and standardised. The involvement of cells from human tissue will help overcome the ethical and scientific disadvantages of the existing mouse models, and provide a real tissue 3D microenvironment to preselect therapeutics for *in vivo* testing in mice. We propose that the concept of a perfused, dynamic culture platform brings us nearer to recapitulating the actual physiological and pathological events in the tumour microenvironment, which will improve the physiological relevance of the current available 3D *in-vitro* tumour models. Moreover, the improved maintenance of patient originated primary GBM cells in the perfusion culture, provides a potential for therapeutic assessment of patient ‘avatars’ of GBM and personalisation of therapy during online treatment of patients.

## Methods

### 2D Cell Preparation

Before cultured in 3D environment, all cell lines were routinely cultured as adherent cells in a humidified incubator at 37 °C with 5% CO_2_. For the detailed information of the medium used for each cell type please refer to Supplementary Information.

### 3D Multicellular cancer spheroid

The perfused 3D cancer spheroids were formed based on the protocol optimised in our lab^15^. Briefly, cell suspensions with 1 × 10^4^/mL cells were added to each well, and the spheroids were allowed to form for 4 days in advance before perfusion culture was applied.

### 3D Sandwich culture

A modified 3D culture protocol was developed to form the Matrigel sandwich structure for both the mono-culture of microtumours and the co-culture of microtumour with angiogenesis network, based on a 3D culture protocol adapted from previous research^36^ and modified in our lab. Briefly, to form the bottom layer of Matrigel, 50 µL pure Matrigel was added into each well which had been pre-chilled on ice, and then the plates were incubated at 37 °C for 30 min, allowing the Matrigel to polymerise. Then for the co-culture, HUVECs were seeded onto the polymerised gel layer at the density of 1.4 × 10^5^ cells/mL, in 250 µL endothelial basic medium (EBM-2, Lonza, UK) supplemented with 2% FBS (Life Technologies, US) and 1% penicillin-streptomycin (PAA). After 4 hours of HUVECs seeding, OVCAR8 cells in 10% Matrigel (v/v) in 250 µL pre-chilled EBM-2 supplemented with 2% FBS and 1% penicillin-streptomycin were added onto the polymerised Matrigel at the density of 5 × 10^4^ cells/mL. The plate was then left at 37 °C to allow the top layer of Matrigel to polymerise. The co-culture system was maintained in EBM-2 supplemented with 2% FBS and 1% penicillin-streptomycin at 37 °C, 5% CO_2_ for at least 24 hours before further drug testing. For the mono-culture of microtumours only, the process was the same as the procedure described in co-culture, except that the only difference was that no HUVECs were added, so cancer cells were cultured alone between the 50 µL bottom layer and 25 µL top layer of Matrigel after the polymerisation process.

### Microbioreactor fabrication

The initial bioreactor was manufactured in our own lab and previously reported^15^. Briefly, poly(dimethylsiloxane) (PDMS) polymers and crosslinking reagent (Sylgard 184 silicone elastomer, Dow Corning) with the ratio of 10:1 were poured into a designed mould and cross-linked at 60 ^o^C overnight. The product bioreactor, a single direction, multiple parallel perfused micro-bioreactor system is shown in Fig. 1. The nutrient rich culture media or tested anti-cancer drugs are individually perfused through inlet tubing by a syringe pump (Harvard Apparatus, US) to the bioreactors, where 3D micro-tissues are housed in the wells with the same size of the wells of 24-well or 96-well plates, depending on the amount of cells required, and the nutrient depleted medium is collected separately through the outlet tubing.

### Real-time monitoring

#### Cell viability assay

For IC50 estimation for a specific model to a certain drug, AlamarBlue (Life Technologies, US) cell viability testing was conducted based on the instructions from the manufacturer. Cells were incubated with EBM-2 containing 10% AlamarBlue for 2 hours at 37 °C. The fluorescence intensity was measured at 560 nm excitation and 590 nm emission using a fluorescent micro-plate reader (WALLAC VICTOR2 1420 multilabel counter model, Perkin Elmer, UK). The inhibition percentage of anti-cancer drug on the viability of cells was calculated as below: Percentage of Inhibition = [cell viability of untreated sample − cell viability of sample treated by anti-cancer drug]/cell viability of untreated sample × 100%. The log-dose dependent responses were then fitted into Sigmoidal functions by GraphPad Prism (GraphPad Software, US) to calculate IC50 estimation.

#### Living cell labelling for co-culture

Cancer cell lines and human umbilical vein endothelial cells (HUVECs) were stained by 5 µM (in PBS) 5-chloromethylfluorescein diacetate (CellTracker Green CMFDA, Molecular Probes, UK) and (5 µM) 5-(and-6-)-(((4-chloromethyl)benzoyl)amino) tetramethylrhodamine (CellTracker Orange CMTMR, Molecular Probes, UK), respectively. The probes were diluted from 10 mM stock solution in DMSO into PBS and incubated with the cells at 37 °C, 5% CO_2_ for 30 minutes. These probes were designed for relatively long-term tracking, lasting for at least three generations of passages and therefore chosen for this study. The images were taken using a fluorescence microscope (Nikon TE-2000, UK) for analysis. The details for microscope parameters are provided in the Supplementary Data.

### End-point assay

#### Sectioned-spheroid fixation and staining for lung cancer multicellular spheroid model

The spheroids were harvested into agarose before being fixed in a 4% paraformaldehyde solution for 24 hours, they were then immersed in 70% ethanol for 24 hours. The spheroids were then embedded in paraffin to a GCP small tissue protocol (Leica TP2200, Germany and ThermoFisher Scientific – Histostar, USA) the FFPE blocks were cut at 4 um + /− 1 um using a Leica Microtome (RM2225, USA) and mounted onto SuperFrost slides (ThermoFisher Scientific Superfrost Plus). The slides were deparaffinised in formalin twice for 3 mins each, dehydrated in 100% ethanol twice for 3 mins each and then rehydrated in an ethanol gradient (70% and 50% ethanol, with 3 mins each). The slides then underwent heat induced epitope retrieval – using a sodium citrate buffer pH 6.0 at 95 deg C for 2 mins they were then cooled on the benchtop. The slides were blocked using an endogenous peroxidise block (Dako Dual endogenous enzyme block – S2003, USA). The antibodies were diluted in ABCAM diluent (ab64211, Abcam, UK) and incubated on the samples for 24 hours at 4 °C, they were then washed in PBS and AlexaFlour-conjugated secondary antibodies were incubated for 30 mins in the dark and the slides were cleaned and mounted with a fluorescent and DAPI mountant (VECTASHIELD H-1200, USA).

#### Whole-culture fixation and staining for neuroblastoma Matrigel sandwich model

Microtumour cultured on their own or in co-culture with the vascular endothelial cells were fixed directly in the Matrigel sandwich for 15 minutes, to maintain the original 3D structures such as the tubule network formed by endothelial cells. For immunofluorescence, cells in 3D culture were stained using a protocol modified for sandwich Matrigel^36^. The samples were first rinsed by PBS – glycine (100 mM glycine in PBS) for three times, then blocking buffer (10% goat serum (Sigma), 1% goat F(ab′)2 anti-mouse immunoglobulin G (Caltag, UK) in staining buffer (PBS supplemented with 0.2% TritonX-100, 0.1% BSA, and 0.05% Tween 20) was incubated on the samples to block the unspecific binding sites. The samples were then incubated with the desired primary antibodies at 4 °C overnight, followed by the incubation in the suitable second antibodies at room temperature for 45 minutes and finally were mounted with diaminophenylindole (DAPI) (Vector Labs, UK). Images were taken and processed on a confocal microscope (Zeiss 710, UK). The details of the antibodies used in immunofluorescence are listed in the Supplementary Data.

#### Extracted-culture fixation and staining for glioblastoma Matrigel embedded model

Due to the thickness and high cell density in the 3D structure of the Matrigel embedded model, a cell recovery protocol was applied to extract the tissue from the Matrigel hydrogel. Briefly, cultures embedded in the Matrigel were rinsed with ice-cold PBS, and the microtumours were extracted by incubation with Matrigel cell recovery solution (BD, UK) for 1–1.5 hour. The mixture of the Matrigel embedded culture and the recovery solution was gently shaken and checked from time to time to ensure Matrigel was fully digested. Then the extracted microtumours were separated from the solution via 150 g centrifuge, and fixed by 4% PFA for 30 minutes. The following procedure was the same as in whole-culture fixation and staining as above.

#### Chemotherapeutic responses

Retinoic acid (10 µM) was added or perfused into the neuroblastoma microtumours in Matrigel sandwich at day 13 counted from the initial starting seeding day (as day 0), and the perfusion rate was designed as: perfusion rate (r) = medium change volume (v)/medium change time (t). For example, for culture time longer than 14 days, in static culture 1000 µL of medium will be changed every 7 days, then the perfusion rate will be: r = 1000 µL/(24 hours × 7 days) = 5.95 µL/hr. For testing time 7–14 days, in static culture 1000 µL of medium will be changed every 3 days, and this will lead to a perfusion rate r = 1000 µL/(24 hours × 3 days) = 13.8 µL/hr. For the supplementary information on ovarian cancer Matrigel sandwich model, Paclitaxel (10 µM) was applied as a classic representative for first-line anti-cancer drugs, after the microtumours were initially cultured for 3 days.

Primary cells were obtained with ethical approval and informed consent from Oxford University Hospitals and all methods were carried out in accordance with relevant guidelines and regulations of the local Research Ethics Committee (REC).

### Statistics

All experiments were repeated three times independently, with each experiment having four parallel samples. Student’s t-test was used to compare the significant difference between different culture methods. P < 0.05 was considered to be significantly different.

## Electronic supplementary material

Supplementary Information
