# Supplementary material for: Perfused Three-dimensional Organotypic Culture of Human Cancer Cells for Therapeutic Evaluation
Source: Sci Rep. 2017 Aug 25;7:9408. doi: 10.1038/s41598-017-09686-0 (PMC5573358; doi:10.1038/s41598-017-09686-0)

Perfused Three-dimensional Organotypic Culture of Human Cancer Cells for Therapeutic Evaluation

Xiao Wan[1], Steven Ball[2], Frances Willenbrock[1], Shaoyang Yeh[3], Nikola Vlahov[1], Delia Koennig[1], Marcus Green[1], Graham Brown[1], Sanjeev Jeyaretna[4], Zhaohui Li[3], Zhanfeng Cui[3], Hua Ye[3], Eric O'Neill[1]*

[1] CRUK/MRC Oxford Institute of Radiation Biology, University of Oxford, ORCRB Research Building, Roosevelt Drive, Headington, OX3 7DQ, UK. [2] Oxford Instruments Nanoscience, Tubney Woods, Abingdon, Oxford, OX13 5QX, UK [3] Institute of Biomedical Engineering, Department of Engineering Science, University of Oxford, Old Road Campus Research Building, Headington, Oxford, OX3 7DQ, UK. [4] Department of Neurosurgery, John Radcliffe hospital, Headington, Oxford OX3 9DU, UK.

* Correspondence to: Dr Eric O'Neill, eric.oneill@oncology.ox.ac.uk

Supplementary information and tables s1-s3.

Figure s1. Perfusion eliminated the necrotic core cause by insufficient nutrient and oxygen supply in 3D multicellular spheroids formed by lung cancer cells H1299. It is interesting that tubulin expression was also increased in the perfusion group.

Figure s2. HUVECS grown on Matrigel under static or perfused conditions as indicated.

Figure s3. **a**. Live cell staining of SY5Y cells (CellTracker Green) and human umbilical vein endothelial cells (HUVECs) (CellTracker Orange) co-cultures on Matrigel. **b,** time course of co-culture over 14 days separated images for Figure 6a. Scale bar: 500µm.

Figure s4. HUVEC and OVCAR8 cells co-cultured in the bioreactor statically at day 14. HUVECs stained with living cell staining CellTracker Orange (red) and OVCAR8 stained with CellTracker Green (ThermoFisher, UK), co-cultured in the bioreactor statically at day 14. Microtumours were co-stained for cleaved caspase - 3 (rabbit anti-human, CST, UK; goat anti-rabbit IgG labelled with Alexa 633). All the samples were fixed as described previously. Scale bar: 100µm.

Figure s5. GBM microtumours were formed by embedding glioblastoma cell line GaMG cells in Matrigel, and then the 3D structures were extracted as described in Materials and Methods, followed by staining with Astrocyte differentiation glial fibrillary acidic protein (GFAP, red fluorescence) and pluripotency marker Nanog (green fluorescence). Scale bar: 100µm.

Table s1. Medium information for 2D cell expansion prior to 3D culture module

Names	Cell types	Basic medium	Supplements
SY5Y (ATCC)	Neuroblastoma cell line	DMEM (Gibco, UK)	10 % (v/v) foetal bovine serum (FBS) (Gibco, UK) and 100 U penicillin/ml and 100 lg streptomycin/ml (Gibco, UK)
GAMG (DSMZ)	Glioblastoma cell line	DMEM (Gibco, UK)	10 % (v/v) foetal bovine serum (FBS) (Gibco, UK) and 100 U penicillin/ml and 100 lg streptomycin/ml (Gibco, UK), 4x NEAA
Human primary glioblastoma cells	Human primary glioblastoma cells	Neural Basal Medium (Gibco, UK)	Neurobasal medium, 3mM glutamine, 1x B27 supplement, 0.5x N2 supplement, 2μg/ml heparin, 50 U/ml penicillin and 50 μg/ml streptomycin, 250μg/ml amphotericin B, 20ng/ml rec (all Gibco), human EGF (Peprotech), 20ng/ml rec human FGF (Peprotech).
OVCAR8 (NIH)	Ovarian cancer cell line	DMEM (Gibco, UK)	10 % (v/v) foetal bovine serum (FBS) (Gibco, UK) and 100 U penicillin/ml and 100 lg streptomycin/ml (Gibco, UK)
H1299 (ATCC)	Lung cancer cell line	DMEM (Gibco, UK)	10 % (v/v) foetal bovine serum (FBS) (Gibco, UK) and 100 U penicillin/ml and 100 lg streptomycin/ml (Gibco, UK)
HUVECs (Lonza UK)	Primary vascular endothelial cells	Endothelial Basal Medium – 2 (Lonza, UK)	Endothelial Growth Medium – 2 (Lonza, UK)

Table s2. Technical comparison of microtumours in this study

Models	Hydrogel used	Polymerisation	Description
Multicellular spheroids	Agarose (60µL)	At room temperature for 30 min	Lower cost; Lack of extracellular matrix
Matrigel sandwich	4-well bioreactor 75µL Matrigel<br>10-well bioreactor 15µL Matrigel	Bottom layer polymerised at 37°C for 30min; then top layer with cell suspension at 37°C overnight.	Medium cost; Laminin-rich extracellular matrix; tubular structure maintained; lower 3D depth
Matrigel embedded	4-well bioreactor 100µL Matrigel<br>10-well bioreactor 25µL Matrigel	At 37°C for 30min	Higher cost; Laminin-rich extracellular matrix; no complicated structure; high 3D depth.

Table s3. List for primary antibodies used in the immunofluorescence microscopy

Functions	Antigen names	Host species	Supplier	Index
Pluripotency markers	SOX2	Mouse anti-human	Millipore (UK)	Fig. 5a; Fig. 6b
	Nanog	Mouse anti-human	Cell Signalling Technology (UK)	Fig. 8; Fig. s2
Neuron differentiation	β – tubulin III	Rabbit anti-human	Biomass Antibodies (US)	Fig. 5a; Fig. 6b; Fig. s1
Astrocyte differentiation	GFAP	Rat anti-human	Thermo-Fisher (UK)	Fig. 8; Fig. s2

Figure s1

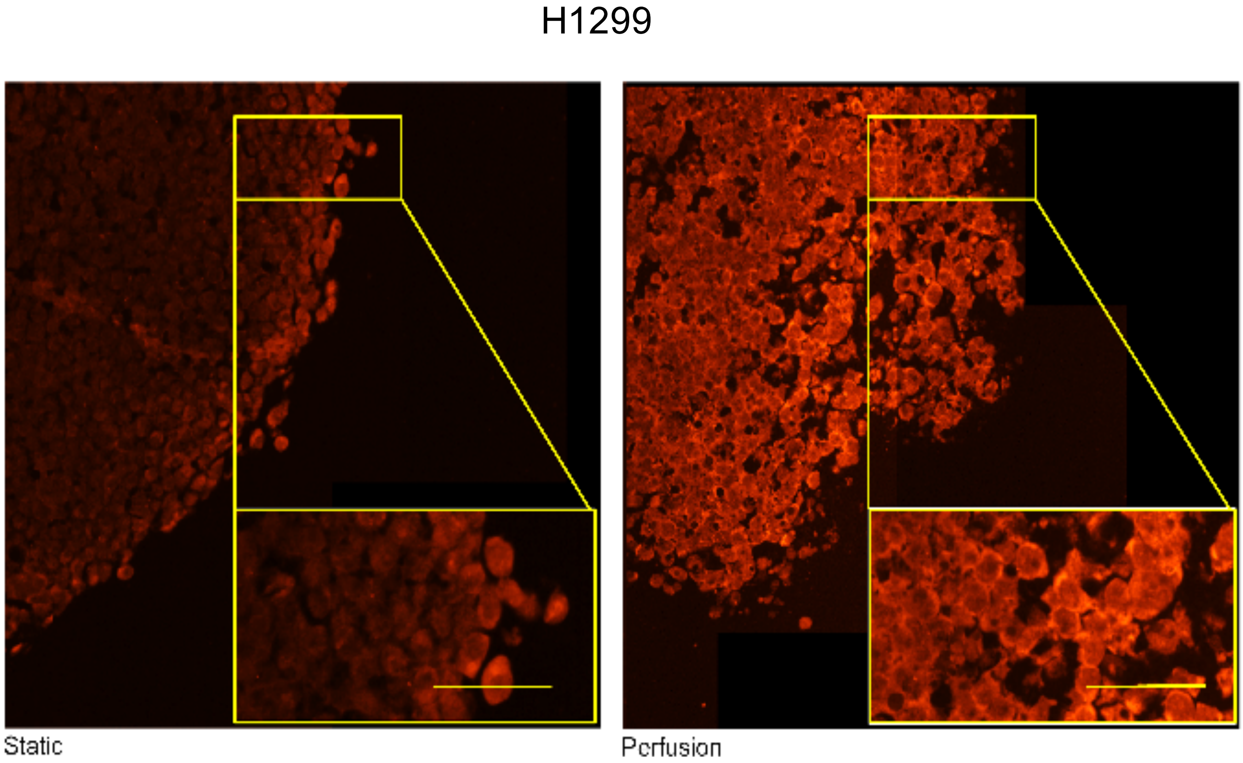

Figure s2

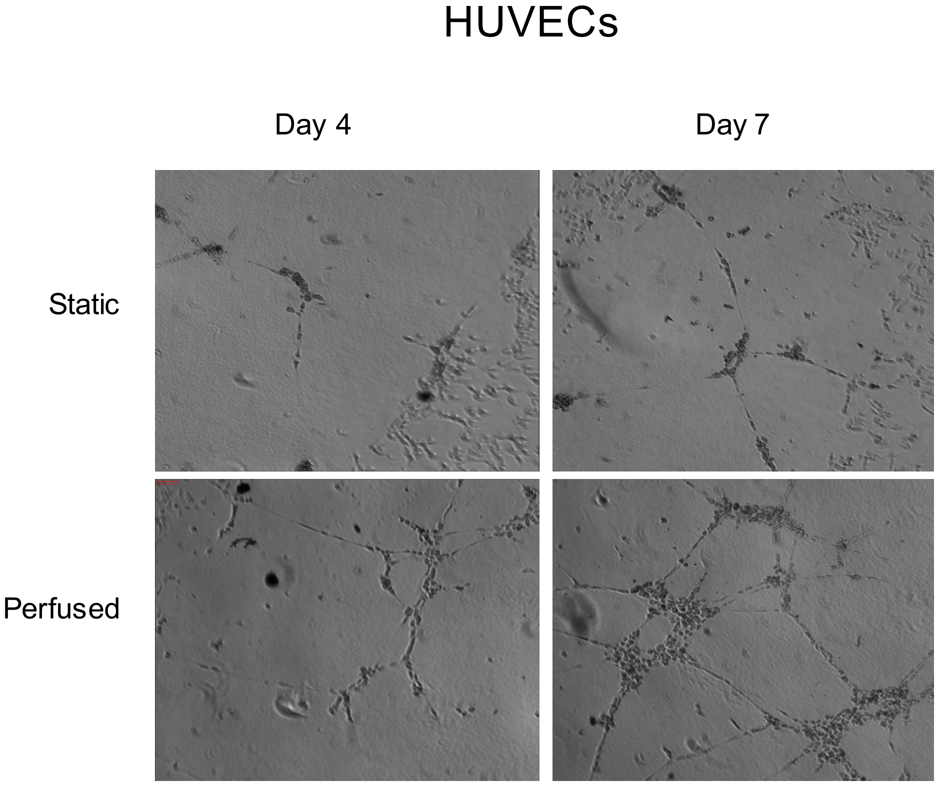

Figure s3

a

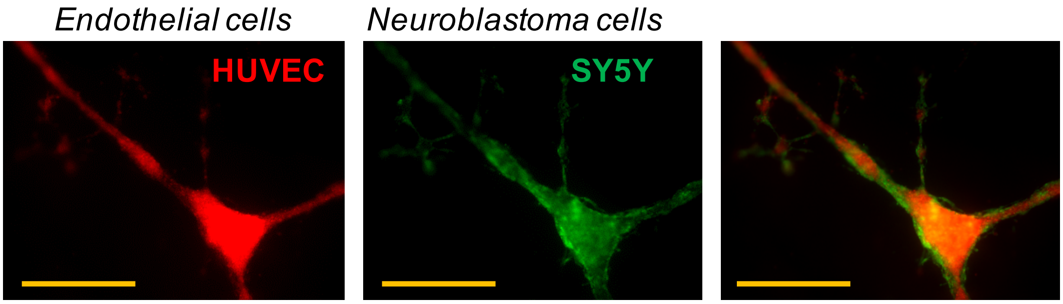
Endothelial cells
Neuroblastoma cells
HUVEC
SY5Y

b

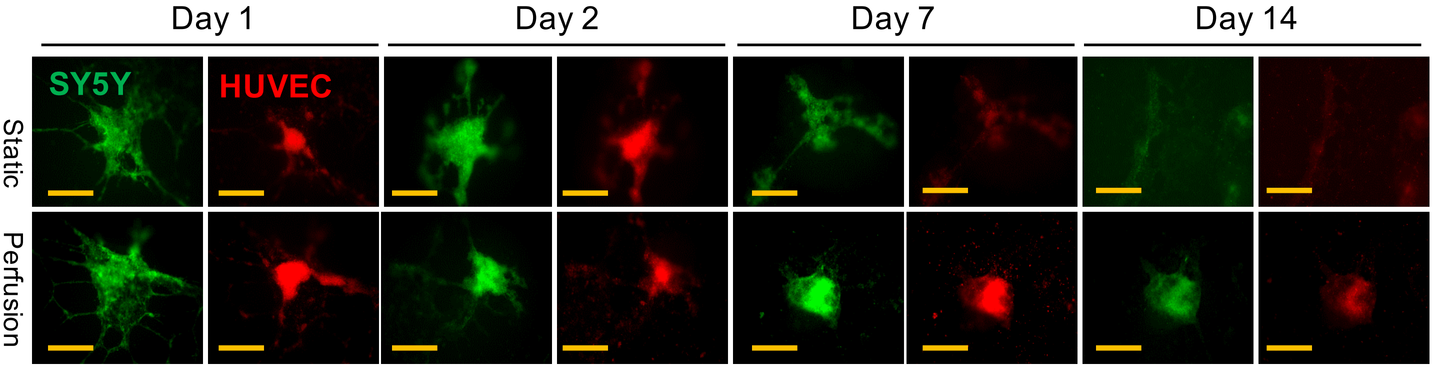
Day 1
Day 2
Day 7
Day 14
SY5Y
HUVEC
Static
Perfusion

Figure s4

HUVEC and OVCAR8 co-cultures

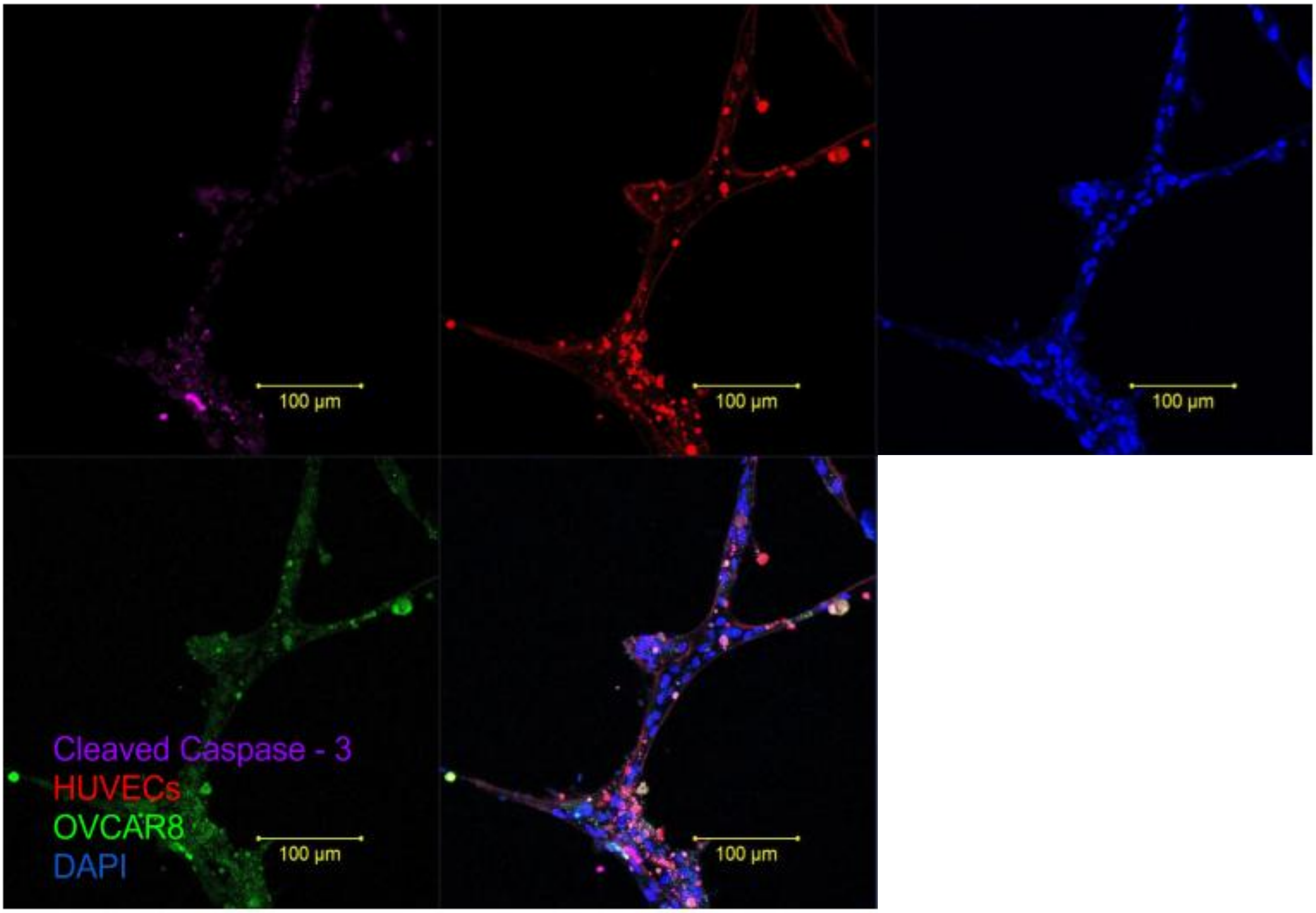

Figure s5

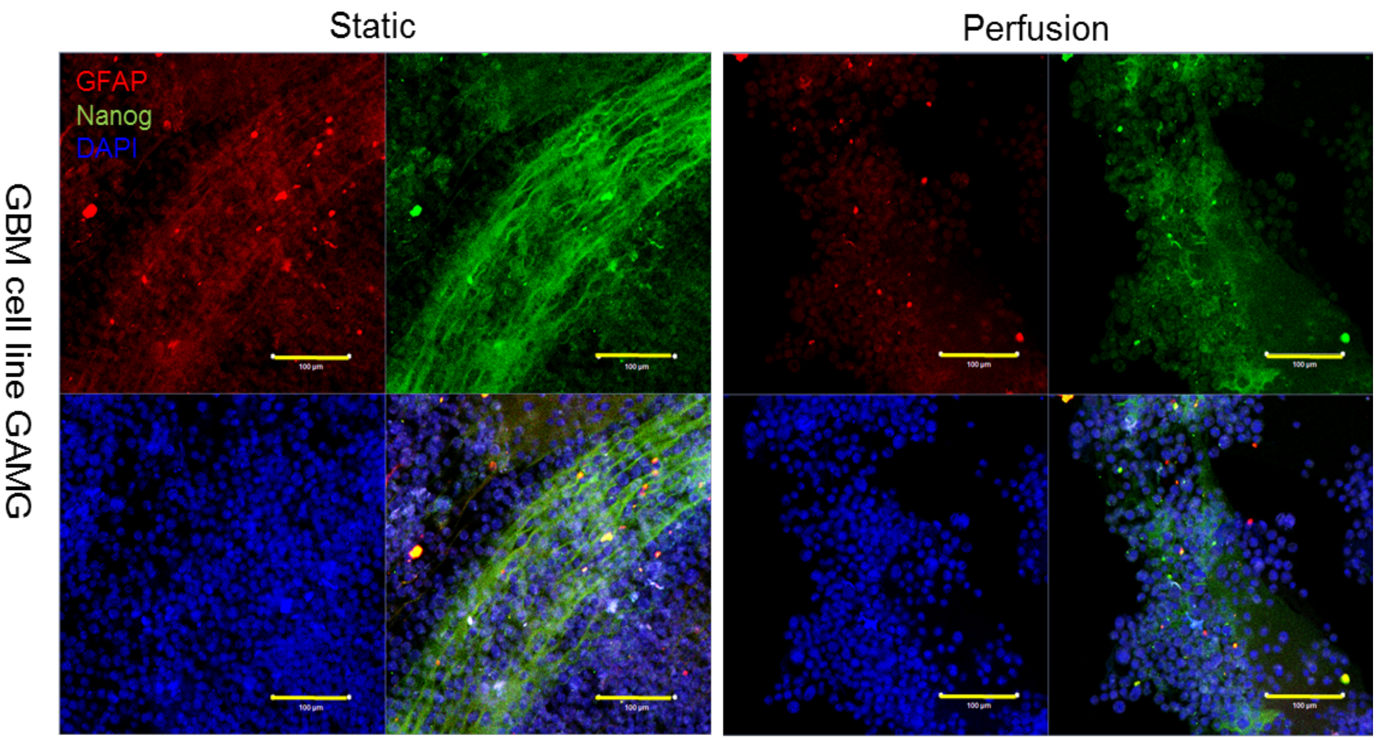

Supplement: Supplementary file 1 — Supplementary Information [file 41598_2017_9686_MOESM1_ESM.pdf]
